# Fibromyalgia in Iraqi patients with asthma and its impact on asthma severity and control

**DOI:** 10.1016/j.amsu.2020.10.019

**Published:** 2020-10-16

**Authors:** Faiq I. Gorial, Manal Abbas Allawerdi, Mustafa Neeama Abd Ali

**Affiliations:** a Rheumatology Unit, Department of Medicine, College of Medicine, University of Baghdad, Baghdad, Iraq; b Rheumatology Unit, Baghdad Teaching Hospital, Baghdad, Iraq; c Respiratory Unit, Department of Medicine, College of Medicine, University of Baghdad, Baghdad, Iraq

**Keywords:** Fibromyalgia, Asthma, Asthma severity, Asthma control

## Abstract

**Background:**

Fibromyalgia (FM) is common with significant impact on patients quality of life. Limited reports on coexistence of FM with asthma.

**Objectives:**

To assess the prevalence of FM in asthmatic patients and its impact on asthma severity and control.

**Patients and methods:**

This case-control study included 103 patients with asthma and 102 apparently healthy controls matched in age and sex. Sociodemographic and clinical characteristics of FM and controls were recorded. FM was diagnosed according to the 2016 revision of American College of Rheumatology criteria. Asthma diagnosis and severity were performed by the pulmonologist according to Global Initiative for Asthma (GINA) guidelines and asthma control was assessed by Asthma Control Test (ACT) score.

**Results:**

The mean age of asthmatic patients was 41.1 ± 12.7 years and for controls was 39.8 ± 12 years (p = 0.453). Females were more prevalent in asthmatic patients and controls although statistically were not significant (p-value = 0.532). Prevalence of FM was significantly more in asthmatic patients compared to controls [18 (17.6%) vs 7 (6.8%), p = 0.018] and asthmatic patients had three folds risk of having FM (ranging from 1.2 to 7.4 times. FM increased the risk of severe asthma by 4.91 folds (P < 0.005). Also, only FMS and glucocorticoids were significant independent predictor of having poor asthma control. FM was significantly and negatively correlated with low ACT score (β standardized regression coefficient = −0.291, p = 0.005).

**Conclusions:**

fibromyalgia was common in asthmatic patients and was significantly associated with more severe and poorly controlled asthma.

## Highlights

• Fibromyalgia (FM) was more common in asthmatic patients compared with controls.
• FM was significantly associated with more severe and poorly controlled asthma.
• Breathing training programs may be useful management in asthmatic patients with FM.

## 1 Introduction

Fibromyalgia (FM) is characterized by chronic pain, fatigue and functional symptoms, without any obvious organic lesion [1]. In general population, the prevalence ranges between 0.2 and 6.6% [2]. Most of the patients are middle aged women; however, it has also been described in children [3,4].

There is still controversy concerning the etiopathogenesis of FM with genetic predisposition, environmental factors and neuromodulation all being considered to be involved in onset and course of the disease [1].

Asthma is a chronic obstructive pulmonary disease with high impact on patients quality of life and diagnosed by history of variable respiratory symptoms and confirmed variable expiratory airflow limitation [5].

Given the progressive increase in the prevalence of FM in recent years, and because of the increasing realization that FM has associations with many other diseases; the coexistence of asthma and FM in a single patient may be plausible [6,7]. This is in addition to that both conditions share some of the associated features like: depression, anxiety, sleep disturbance, cognitive impairment and obesity in addition to being more prevalent in female gender.

The study was designed to assess the prevalence of FM in asthmatic patients and its impact on asthma severity and control.

## 2 Patients and methods

### 2.1 Study design

This case-control study was conducted at Respiratory and Rheumatology Units in Baghdad Teaching Hospital and Dowaly Private Hospital from August 2018 till June 2019. Ethical approval was obtained from the Ethical committee in Department of Medicine, College of Medicine, University of Baghdad in accordance with the Declaration of Helsinki with ethical approval reference no: 2 with a date of January 15, 2020 and a singed informed consent was obtained from each participant. This study has been done and reported in line with the STROCSS criteria [8].

### 2.2 Participants

Eligible patients were ≥18 years old of either gender, had established diagnosis of asthma according GINA guidelines [9], Patients were excluded if they had connective tissue disease, inflammatory arthropathy, osteomalacia, thyroid disease, established malignancy or pregnancy by history and clinical examination. Controls selection was from apparently health individuals collected from the relatives of other patients attending rheumatology outpatients’ clinic. And were comparable in age and sex to patients.

### 2.3 Data collection, entry, and evaluation

All the participants in the study were seen and examined consecutively by the same expert rheumatologist doctor for FM and same expert pulmonologist for asthma diagnosis. Data collection from asthmatic patients and controls were performed using a clinical research form sheet containing questionnaire through direct face to face interview.

All the participants were assessed for their sociodemographic data: age, gender, educational level, occupation, body mass index (BMI = weight/height^2^) and for their smoking status, duration of asthma, and medication history were documented. Blood investigations (including complete blood count, blood biochemistry, thyroid function test, vitamin D level and serological and inflammatory markers), radiographs and/or other tests were performed for both groups when it was indicated according to the clinical situation and physical examination findings.

We assessed FM using the 2016 revision of American College of Rheumatology (ACR) criteria was used for the diagnosis of FM [10]. Full history was taken from both groups, and all participants were asked about the presence of generalized pain (at least 4 out of 5 regions) and widespread pain index WPI (0–19) for at least three months duration. The level of severity of fatigue, un-refreshing sleep and cognitive dysfunction during the last week were scored from 0 to 3. Migraine or tension like headache, depression; which was assessed using Diagnostic and Statistical Manual of Mental Disorders, Fifth Edition (DSM-5) questionnaire [11] and lower abdominal cramps that occurred during the previous 6 months were evaluated and scored if present or absent as 0 or 1, respectively. According to the summation of these scores the symptoms severity score (SSS) was calculated. Totally 12 or more of FMS symptoms or polysymptomatic disease scale (PDS); which is calculated by summation of WPI and SSS (PDS = WPI + SSS), is required for a patient to be diagnosed with FMS.

Asthma diagnosis and severity assessment was performed by pulmonologist according to Global Initiative for Asthma (GINA) guidelines for asthma diagnosis [8]. The level of symptom control in the last month was assessed by the investigator for all patients using Asthma Control Test (ACT) [12]. Asthma Control Test (ACT) is a patient completed questionnaire with 5 items assessing the symptoms of asthma during the last 4 weeks regarding daytime and nocturnal symptoms, use of on need (rescue) medications and the effect of asthma on daily activity/school or work attendee. Each item has 5 response options corresponding to Ref. [[1], [2], [3], [4], [5]] scores then the scores of all 5 items are summed to yield a score ranging from 5 to 25. Score results from Ref. [[5], [6], [7], [8], [9], [10], [11], [12], [13], [14], [15], [16], [17], [18], [19]] indicates poorly controlled asthma [[20], [21], [22], [23], [24]], indicates on target and [25] indicates well controlled asthma.

### 2.4 Statistical analysis

A total sample size of at least 183 participants (92 patients and 91 controls) was needed to get a medium effect size of 30% with a significant α error probability of 0.05 and a statistical power (1-β error probability) of 90%. Statistical software SPSS v 24 (IBM, New York, NY, USA). Was used for analysis. Kolmogorov Smirnoff test was used to assess normality of continuous variables. Data was expressed as mean ± SD for normally distributed continuous variables and numbers (percentages) for categorical variables. Student *t*-test was used to find the difference between normally distributed continuous variables and Chi square test for categorical variables. Ordinal regression analysis was used to assess the effect of FMS and other baseline characteristics on asthma severity and multiple linear regression analysis was used to assess the effect of FMS and other baseline characteristics on ACT score. P < 0.05 was considered statistically significant.

## 3 Results

A total of 205 individuals were included in this study, of them 102 asthmatic patients and 103 apparently healthy controls. The mean age of asthmatic patient was 41.1 ± 12.7 years and that of controls was 39.8 ± 12.0 years (p-value = 0.453). Females were more prevalent in asthmatic patients and controls (p-value = 0.532). The mean BMI in asthmatic patients was 28.3 ± 6.6 kg/m^2^ and in controls was 28.2 ± 6.9 kg/m^2^ (p-value = 0.881). This means that age, gender and mean BMI were not significant sociodemographic confounders in the study as shown in Table 1.

**Table 1:** Baseline characteristics of asthmatic patients and controls.

Variables	Asthmatic patients n = 102	Controls n = 103	p-value
**Age (years), mean ± SD**	41.1 ± 12.7	39.8 ± 12.0	0.453
**Female, n (%)**	52 (51.0%)	57 (55.3%)	0.532
**BMI (kg/m**^**2**^**), mean ± SD**	28.3 ± 6.6	28.2 ± 6.9	0.881
Asthma duration (years), median (range)	7.5 (3.0–20.0)		
Asthma severity, n (%)			
Mild	40 (39.2%)		
Moderate	25 (24.5%)		
Severe	31 (30.4%)		
Very severe	6 (5.9%)		
ACT score, mean ± SD	15.5 ± 6.2		
Drugs used, n (%)			
GC	71 (69.6%)		
Beta – agonist	80 (78.4%)		
Leukotriene agonist	11 (10.8%)		

BMI: body mass index, n: number, SD: standard deviation, ACT: asthma control test, GC: glucocorticoids, n: number, P < 0.05 is significant.

**Fig. 1:**
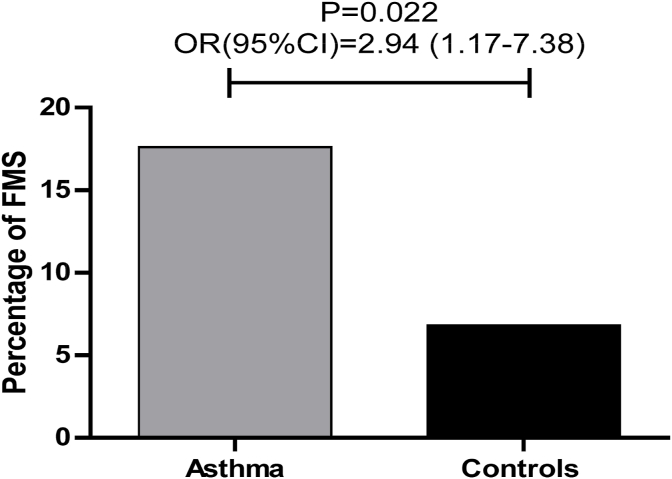
Prevalence of fibromyalgia in asthma and controls.

Prevalence of FM was significantly more in asthmatic patients compared to controls [18 (17.6%) vs 7 (6.8%), p = 0.018] and asthmatic patients had three folds risk of having FM (ranging from 1.2 to 7.4 times) as shown in Fig. 1.

Ordinal regression analysis revealed that FM increased the risk of severe asthma by 4.91 folds (P < 0.005) (Table 2) And on multiple linear regression analysis to assess the impact of FMS on ACT score after controlling the effect of other covariate as confounders showed that only FMS (β = − 0.2906, p = 0.005) and glucocorticoids (β = − 0.3221, p = 0.002) were significant independent predictor of having poor asthma control. FMS was significantly and negatively correlated with low ACT score (Table 3).

**Table 2:** Ordinal regression analysis to assess the impact of baseline characteristic and FM on asthma severity.

Predictor	p	Odds ratio	95% Confidence Interval Lower	Upper
FM present	0.005	4.91	1.641	15.59
Age	0.857	1.00	0.969	1.04
Females compared to males	0.985	1.01	0.431	2.35
BMI	0.004	1.10	1.034	1.18
Asthma duration	0.498	1.01	0.978	1.05
Corticosteroids users	0.033	2.63	1.099	6.55
Leukotriene users	0.342	1.88	0.504	7.10

FM, fibromyalgia; BMI, body mass index.

**Table 3:** Multiple linear regression analysis to assess the impact of FM and baseline characteristics on asthma control score.

Predictors	Standardized regression coefficient β	P
FM	−0.2906	0.005
Age	−0.005	0.963
Gender	0.1068	0.308
BMI	−0.1613	0.122
Education Level	0.1636	0.117
Occupation	0.1152	0.272
Asthma duration	−0.1843	0.077
Corticosteroid users	−0.3221	0.002
Beta agonist users	0.1185	0.258
Leukotriene receptor antagonist users	−0.1504	0.150

R^2^-adjusted = 0.26.

P of the model<0.0001.

## 4 Discussion

This study evaluated the prevalence of FM in asthmatic patients according to the new modified 2016 ACR classification criteria of FM and assessed its effect on asthma severity and controls. It showed that prevalence of FM in patients with asthma was significantly higher compared to controls and asthmatic patients with FM were significantly more severe and poorly controlled.

It is possible that the inflammatory process of asthma can affect the development or expression of other conditions. Based on a hypothesis that the origin of all pain is inflammation and inflammatory response; van West and Maes hypothesized that FMS is an inflammatory disorder accompanied by changes in the neuroendocrine-immune system, although, the sources of inflammation triggering FMS remains to be elucidated [13,14]. In addition, It has been proposed that FMS is due to neurogenic inflammatory response to allergens, infectious agents, chemicals or emotional stress, all of which are also known triggers for asthma symptoms [13]. These, and the known increased in occurrence of pain, sleep disorders, depression and fatigue in asthmatic patients, may explain the relationship between both conditions.

Furthermore, FMS has numerous, systemic biological anomalies and is polysymptomatic. For functional disorders, many theories have been proposed. This particular pathophysiology can be associated with abnormality associated with immune, neurologic and endocrine functions [[14], [15], [16], [17], [18]]. The second type of theory is that a common psychological process, including somatization (i.e. Somatoform disorder), or some kind of cognitive disturbance, triggers all the functional disorders, like FM. In a third, commonly-called biopsychosocial interpretation, psychological and biological mechanisms are merged in one model to describe both biological and psychological condition abnormalities [19,20].

The theory of functional disease symptoms and asthma extra-pulmonary symptoms having similar etiology indicates two predictions. For a group of patients with extreme asthma, the relative frequency of the multiple extra-pulmonary symptoms should be close to that of people with FMS diagnoses. Second, in asthma patients with extra-pulmonary symptoms, the degree of similarity should be increased. Previous investigations revealed that the pattern of symptoms in functional disorder groups is closer to that of fibromyalgia as symptom frequency increases (i.e. relative frequency of symptoms) [21]. The symptoms of people with extreme asthma extra-pulmonary should, when the number of symptoms extra pulmonary rises, be approximate to fibromyalgia symptoms.

Therefore, the high FM in asthmatic patients may be related to the neurogenic inflammatory response to allergens, infectious agents, chemicals or emotional stress which are also known triggers for asthma symptoms. In addition to the increased symptoms of pain, sleep disorders, depression, and fatigue in asthmatic patients [22]. Moreover, the Presence of sleep disturbance in asthma may lead to frequent awakening, early awakening, and non restorative sleep which is a preceding and pathogenic factor the development of FM. Also the Kinine release in asthma which is a potent bronchoconstrictor and one of pain mediators with The stress and psychological factors may possibly release proinflammatory cytokines and trigger FM [23].

In the current study, FM significantly increased the risk of having severe asthma by about five folds (OR = 4.91, P < 0.005) and there was a significant negative correlation between FM and asthma control. These findings are clinically important and may indicate that early diagnosis and treatment of FM can subsequently decrease severity of asthma and improve controlling of asthma and health related quality of life.

Similarly, a precious study observed that asthma patients with concomitant fibromyalgia had alterations in the perception of dyspnea being hyperperceivers during the bronchial provocation test. These alterations in the perception of airway obstruction may explain, at least in part, uncontrolled asthma found in these patients. In addition, emotional status was an independent factor determining the degree of dyspnea in patients with different grades of stable asthma. Accordingly, the high rates of emotional disorders in patients with FM may contribute to high dyspnea scores [[24], [25], [26]].

Hyland et al. [27] tested the similarity in type and etiology between the extra‐pulmonary symptoms of severe asthma and the symptoms of FM and they observed that Patients with severe asthma have numerous extra‐pulmonary symptoms similar in type and pattern to the symptoms of FM which supported the hypothesis that functional disorders and extra‐pulmonary asthma symptoms have a common complexity or network etiology and suggested that evidence based behavioural interventions for FM may be helpful for patients with severe asthma reporting extra‐pulmonary symptoms.

Martinez-Moragon Eva evaluated the characteristics of asthma in patients with concomitant FM and assessed whether FM is an independent factor of asthma severity that influences poor asthma control and they concluded that FM was associated with poorly controlled asthma [7].

On the other hand, in a study of 157 consecutive patients with asthma, the clinically relevant hyperventilation was a common feature among them [28]. Patients with hyperventilation experienced more exacerbation episodes requiring admission to the emergency department and scored significantly higher in subscales of the asthma symptoms. Accordingly, it may be speculated that clinically relevant hyperventilation may also contribute to alterations in the perception of dyspnea in asthma patients with FM. A link has been suggested between symptoms of hyperventilation, hyperperception of dyspnea, lower perceived quality of life and anxiety in asthma patients [[29], [30], [31], [32], [33]] but evidence of the coexistence of these disorders in patients with asthma and FM is inconclusive.

Another observation of note in the present study was that asthmatic patients using corticosteroid were positively and significantly correlated with the asthma severity. Similar findings were reported by other studies which concluded that corticosteroids play an important role in the long term management of severe asthma [[34], [35], [36]].

The current study has some limitations that should be taken into account when interpretation of our findings. Both patients and control groups are convenience samples, Small sample size study, lack of follow up visit, being cross sectional observational study, and finally questionnaire based diagnosis of FM and asthma severity and control may overestimate the diagnosis.

## 5 Conclusions

FM was more common in asthmatic patients compared with controls and was significantly associated with more severe and poorly controlled asthma. The stress, anxiety, and depression in patients with asthma and fibromyalgia may explain the lower asthma control with high incidence of dyspnea and hyperventilation. However, the use of questionnaires based on symptoms of both FM and asthma may overestimate asthma severity, with the risk of overtreatment if the true level of asthma control is underestimated. The early diagnosis of FM and asthma may help in early treatment and prevention of severe adverse consequences of FM and asthma and subsequently improving patients quality of life. Early interventions such as breathing training exercises programs may be useful management in asthmatic patients with FM. Education, stress management, and aerobic exercises can help the patients with FMS and asthma to cope with their symptoms and to improve activities of daily living. Finally, a larger longer prospective case control multicenter follow up study is needed to validate our results and to look for the cause and relationship of the both relatively common conditions in the community are suggested.

## Funding

None.

## Authors’ contribution

All authors (F·I.G, M.A. A., and M. N.A.A) contributed in concept or design of the study, data collection, data analysis or interpretation, writing the paper, and approval of the final version of the paper.

## Guarantor

Faiq I. Gorial

## Consent

All patients signed written informed consent for participation in the study.

## Ethical Approval

The local scientific ethics committee of Department of Medicine, College of Medicine, University of Baghdad approved the study protocol with ethical approval reference no: 2 with a date of 15/01/2020

### Provenance and peer review

Not commissioned, externally peer reviewed.

## Declaration of competing interest

None.

## Appendix A Supplementary data

Application Application
